# PFP@PLGA/Cu_12_Sb_4_S_13_-mediated PTT ablates hepatocellular carcinoma by inhibiting the RAS/MAPK/MT-CO1 signaling pathway

**DOI:** 10.1186/s40580-021-00279-2

**Published:** 2021-10-04

**Authors:** Tianxiu Dong, Jian Jiang, Hao Zhang, Hongyuan Liu, Xiaomeng Zou, Jiamei Niu, Yingxuan Mao, Mingwei Zhu, Xi Chen, Zizhuo Li, Yaodong Chen, Chunying Shi, Xiuhua Yang

**Affiliations:** 1 Department of Abdominal Ultrasound, The First Affiliated Hospital of Harbin Medical University, Harbin, 150001 China; 2 Department of Medical Imaging, Heilongjiang Provincial Hospital, Harbin, 150001 China; 3 Department of Ultrasonic Imaging, First Hospital of Shanxi Medical University, Taiyuan, 030001 China; 4 Department of Radiology, The First Affiliated Hospital of Harbin Medical University, Harbin, 150001 China

**Keywords:** Thermal ablation, Photothermal therapy, Nanocapsule, Hepatocellular carcinoma

## Abstract

Hepatocellular carcinoma (HCC) is one of the most malignant tumors in the world, and patients with HCC face a poor prognosis. The conventional therapeutic strategies for HCC have undergone a challenge-riddled evolution owing to side effects and unsatisfactory efficacy. Here, aiming to provide a new method of HCC elimination, we formulated a novel multifunctional nanocapsule (PFP@PLGA/Cu_12_Sb_4_S_13_, PPCu) with applications in contrast-enhanced ultrasound imaging (CEUS) and photothermal therapy (PTT). These PPCu were successfully constructed with an average diameter of 346 nm (polydispersity index, PDI = 0.276). The reinforced contrast ratio of these PPCu was determined by CEUS, revealing their promising applications in image-guided monitoring of HCC treatment. Furthermore, the excellent photoabsorption and biocompatibility indicated by organ H&E staining indicated that PPCu meet quality expectations for use as photothermal transduction agent (PTA). PPCu treatment at 50 °C and higher temperatures efficiently repressed the proliferation, induced the apoptosis and decreased the motility of HCC cells. These effects might have been results of RAS/MAPK/MT-CO1 signaling pathway inhibition. In summary, PPCu were constructed to integrate CEUS and PTT successfully into therapy, which can lead to HCC elimination through RAS/MAPK/MT-CO1 signaling pathway repression.

**Supplementary Information:**

The online version contains supplementary material available at 10.1186/s40580-021-00279-2.

## Introduction

Hepatocellular carcinoma (HCC) is the fifth most common type of malignant tumor and the second leading cause of cancer-related mortality of men worldwide [1], and HCC treatment has undergone a challenge-riddled evolution [2]. Surgery, chemotherapy and radiotherapy, as conventional cancer therapeutic strategies, have led to unsatisfactory outcomes or severe side effects. Moreover, most patients with HCC are accompanied with cirrhosis and are diagnosed in the middle or late stage of HCC, resulting in an unsatisfactory surgical resection rate [3]. Tremendous efforts have been made to explore better strategies for HCC treatment. Ablation technology, as a form of hyperthermia-induced treatment, achieved through radiofrequency, microwaves, high-intensity focused ultrasound or lasers, has been a treatment candidate because of its advantages, such as noninvasiveness, operation simplicity, and facile repeatability, and most important, it has shown remarkable curative effects in solid tumor treatment [4–8].

Photothermal therapy (PTT), a laser-induced hyperthermic treatment, has acquired a stellar reputation for cancer ablation when increases in local temperature trigger cell death through PTA [9–11]. Hence, the choice of external PTA is of great importance. Conventional PTAs, such as gold nanorods, graphene and carbon nanotubes, exhibit unsatisfactory photothermal conversion efficiency, leading to suboptimal cancer elimination [12–16]. Novel hydrophobic Cu_12_Sb_4_S_13_ nanoparticles were synthesized by Song et al. and have been used in various applications, such as interfacial water evaporation, thermodestruction of the pathogenic bacteria *E. coli* O157:H7 and simulation of solar salt formation [17]. In addition, compared with other PTAs, including carbon nanotubes, MoO_3-x_ and Au clusters, Cu_12_Sb_4_S_13_ nanoparticles possess much higher light-harvesting ability. In this study, we formulated PFP@PLGA/Cu_12_Sb_4_S_13_ nanocapsules (PPCu) via a double emulsion evaporation process. Cu_12_Sb_4_S_13_ nanoparticles were incorporated onto the surface of poly(lactic-co-glycolic acid), (PLGA, a polymer with excellent biocompatibility) nanocapsules, the center of which was loaded with perfluoropentane (PFP, a perfluorocarbon). Perfluorocarbons exhibit superb characteristics for achieving extremely high oxygen solubility and biocompatibility, are widely used in the clinic in contrast-enhanced ultrasound imaging (CEUS) and enhance the therapeutic efficacy of radiotherapy and photodynamic therapy [18, 19]. The liquid PFP core undergoes a phase transition from liquid to gas under low-intensity focused ultrasound (LIFU) to enable CEUS [20, 21]. After PLGA encapsulation, the PPCu exhibit a preferable photothermal temperature with increasing light-harvesting ability and satisfactory biocompatibility.

Increasing evidence has demonstrated that metabolic alterations play critical roles in cancer progression previously equated with the “Warburg effect” [22–24]. Recently, the Warburg effect has been revisited, with increasing attention directed to the central role of mitochondria in cancer therapy. Numerous studies have shown that the cellular functions of mitochondria primarily include the production of energy, regulation of redox states, promotion of cell death, and supply of biosynthesized metabolites [25, 26]. Therefore, mitochondria enable cancer cell survival, growth and metastasis in a pathological environment by regulating biosynthetic and bioenergetic supplies. Hence, targeting mitochondria-associated signaling pathways has emerged as a potential approach to suppress HCC evolution [27].

In this study, by integrating CEUS and PTT, we found that PPCu-mediated PTT efficiently ablated HCC. To explore the mechanisms of PTT, we assessed the effects of multiple hyperthermia-inducing temperature gradients on HCC. The results revealed cancer elimination under hyperthermic conditions established at temperatures higher than 50 °C, indicating increased apoptosis and inhibited metastasis of cancer cells. Furthermore, RNA sequencing was carried out to confirm mitochondrial metabolism alterations under hyperthermic stress. Briefly, PPCu-induced PTT eliminated HCC tumors through the RAS/MAPK/MT-CO1 signaling pathway, leading to mitochondrial dysfunction and subsequent apoptosis.

## Methods and materials

### Cell lines and reagents

Huh7 and HCCLM3 human HCC cells were supplied by the Institute of Chinese Academy of Science, China. HepG2 cells were supplied by the American Type Culture Collection (Manassas, VA, USA). All these cells were cultured in Dulbecco’s modified Eagle’s medium (DMEM) supplemented with 10% fetal bovine serum and 1% antibiotics at 37 °C in 5% CO_2_. Cu_12_Sb_4_S_13_ nanoparticles were kindly provided by Chongshen Guo from the Key Laboratory of Microsystem and Microstructure in the Harbin Institute of Technology. mPEG5000-PLGA (MW: 15 kDa) was purchased from Jinan Daigang Bio-Technology, Inc. (Jinan, China). Perfluoropentane (PFP) and poly(vinyl alcohol) (PVA, 99% MW = 30,000–70,000 Da) were obtained from Sigma–Aldrich (St. Louis, MO, USA). Chloroform (CHCl_3_) and isopropyl alcohol were obtained from Chongqing East Chemical Industry Ltd., Co. (Chongqing, China). A Calcein-AM/ propidium iodide (PI) double staining kit was purchased from Dojindo Laboratories (Kumamoto, Japan). PD98059 was obtained from MCE (New Jersey, USA).

### Synthesis of the PPCu

In this work, Cu_12_Sb_4_S_13_ nanoparticles were synthesized through a hot-injection method in which oleylamine and glycerol were used as the reaction system [17]. Hence, the sample is highly hydrophobic and disperses readily in dichloromethane. PPCu were prepared through the double emulsion strategy [28, 29]. Briefly, 50 mg of mPEG5000-PLGA and 30 mg of Cu_12_Sb_4_S_13_ nanoparticles were dissolved in 4 mL of dichloromethane. After addition of 600 μL of PFP to the system, sonication was performed at 100 W for 3 min (on:off = 1:1) in an ice bath. Then, 8 mL of PVA (4% w/v) was poured into the emulsion and homogenized with sonication for another 3 min. Afterward, 10 mL of isopropanol (2% v/v) was added for solidification. Finally, the PPCu were centrifuged, washed and dissolved in 2.5 mL of deionized water for storage at 4 °C. Cu_12_Sb_4_S_13_@PLGA and PFP@PLGA nanoparticles were synthesized through a similar process except that in the former, PFP was replaced with deionized water, and in the latter, Cu_12_Sb_4_S_13_ was not added to the mixture.

### Vaporization and CEUS of the PPCu

To examine the acoustic droplet vaporization (ADV), a LIFU instrument was used to stimulate the PPCu at the following intensities: 0 W/cm^2^, 3 W/cm^2^ and 8 W/cm^2^. Then, the change in PPCu was observed through an optical microscope (Olympus DP70, Canada). For the observation of CEUS scans, degassed water and PPCu (5 mg/mL) were loaded into a gel mold (3% agar w/v in distilled water), and subsequent US with an intensity of 8 W/cm^2^ released by the LIFU probe was used to elicit ADV. B-mode imaging and CEUS of the PPCu were realized using a Philips iU Elite Ultrasound System (Philips Healthcare, Amsterdam, The Netherlands) with a linear probe. For in vivo experiments, PPCu were injected into veins, and US imaging was performed 24 h later.

### Real-time PCR

Total RNA was extracted from cultured cells by TRIzol. Reverse transcription was performed using a reverse transcription kit (TaKaRa) after RNA quantification. Real-time PCR was performed using SYBR Green PCR Master Mix (Life Technologies, Carlsbad, CA, USA). Expression levels of target genes were normalized to the level of β-actin and calculated according to the 2^−ΔΔCt^ method. The following primer sequences were used:

MT-CO1 F: CGCCGACCGTTGACTATT,

MT-CO1 R: GCCAAAGCCTCCGATTATG;

MT-CO2 F: CGACTACGGCGGACTAATCT,

MT-CO2 R: GGTCGTGTAGCGGTGAAAGT;

MT-CO3 F: CACTCCATAACGCTCCTCAT,

MT-CO3 R: GTGTGGTGGCCTTGGTATGT;

MT-ND4 F: AGCCACATAGCCCTCGTAGT,

MT-ND4 R: TGTGAGTGCGTTCGTAGTTTG;

MT-CYB F: CACATCAAGCCCGAATGA,

MT-CYB R: CAGGTTAGAATGAGGAGGTCTG; and

ACTB-F: GGGAAATCGTGCGTGACATT,

ACTB-R: GGAACCGCTCATTGCCAAT.

### Western blotting

Cell proteins were extracted with RIPA buffer containing protease and phosphatase inhibitors. After 80–120 V electrophoresis, proteins were transferred to a polyvinylidene fluoride membrane (Invitrogen). After blocking with 5% skim milk, the membrane was incubated with antibodies. Antibodies against p-AKT, AKT, p-ERK, and ERK were obtained from Cell Signaling Technology (Beverly, MA, USA). Antibodies against KRAS, E-cadherin, N-cadherin, Vimentin, ZEB2 and β-actin were supplied by Proteintech Group (Wuhan, China). An antibody against MT-CO1 was purchased from ABclonal (Wuhan, China). Protein bands were visualized with an enhanced chemiluminescence (ECL) reagent (Pierce, Rockford, IL, USA).

### Cell migration and invasion assays

For cell migration assays, 2–4 × 10^4^ cells in 300 μL of serum-free medium were seeded in the upper chamber of a Transwell insert (8 μm pore size) (BD Biosciences, San Jose, CA, USA). The lower chamber was filled with 700 μL of complete DMEM. The cells were incubated in this system for 48 h at 37 °C. Then, methanol and 0.5% crystal violet were used to fix and stain the cells that had migrated through the membrane. The cell invasion assay was performed in the same way as the migration assay except the membrane was coated with Matrigel. Finally, photographs of the migratory and invasive cells were taken at 100× magnification. Each experiment was performed in triplicate.

### Apoptosis assays

Apoptosis was detected using an Annexin V-FITC/PI apoptosis detection kit (4A Biotech, Beijing, China). After treatment under hyperthermic conditions for 48 h, HCC cells were washed and stained with Annexin V-FITC/PI following the manufacturer’s instructions. Finally, apoptosis was evaluated by flow cytometry (FACSCalibur, BD Biosciences).

### Animal experiments

Male BALB/c nude mice (4–5 weeks old) were obtained from Vital River Laboratory Animal Technology Co., Ltd. (Beijing, China) and housed in a pathogen-free environment according to institutional animal care guidelines. The animal experiment scheme was reviewed and approved by the Ethics Committee of Harbin Medical University. For a subcutaneous HCC model, all mice were subcutaneously injected in the right flank with 4 × 10^6^ HepG2 or Huh7 cells (in 200 µL of PBS) that had been heated in a 50 °C water bath for 10 min, and the same number of untreated HepG2 or Huh7 cells were heated and injected into the left flank to generate a control. For PTT assays, HepG2 cells were injected only into the left flank of the mice. A lung metastasis assay was performed after mice were subjected to tail vein injections of 4 × 10^6^ cells in 100 μL of PBS.

### Ultrasound imaging and magnetic resonance imaging

Ultrasonography, including B-mode ultrasonography, color Doppler flow imaging (CDFI), color power angiography (CPA) and ultrasonic elastosonography (USE), was performed with a Philips iU Elite Ultrasound System (Philips Healthcare, Amsterdam, The Netherlands). MRI was carried out with a Philips Achieva 3.0 T TX MRI System. The specific imaging sequences included T1WI, T2WI, T2-SPIR and T2-FLAIR.

### Immunohistochemistry and TUNEL assay

Tumor tissues excised from HCC tumor-bearing mice were fixed with formaldehyde and embedded in paraffin. Then, tumor sections were stained with MT-CO1 and Ki-67 antibodies. The tumor tissues were also labeled with TUNEL stain (Wanleibio, Shenyang, China). Images were obtained with an Olympus BX53 fluorescence microscope (Olympus, Tokyo, Japan). The results were analyzed using Image-Pro Plus 6.0 software.

### Statistical analysis

Data are presented as the mean ± standard deviation (SD). Statistics were analyzed using the GraphPad Prism software package (v. 4.02; San Diego, CA). Differences were evaluated by Student’s *t*-test or ANOVA. Significance is represented by a *p* value < 0.05.

## Results

### Preparation and characterization of a novel PTT agent

**Fig. 1:**
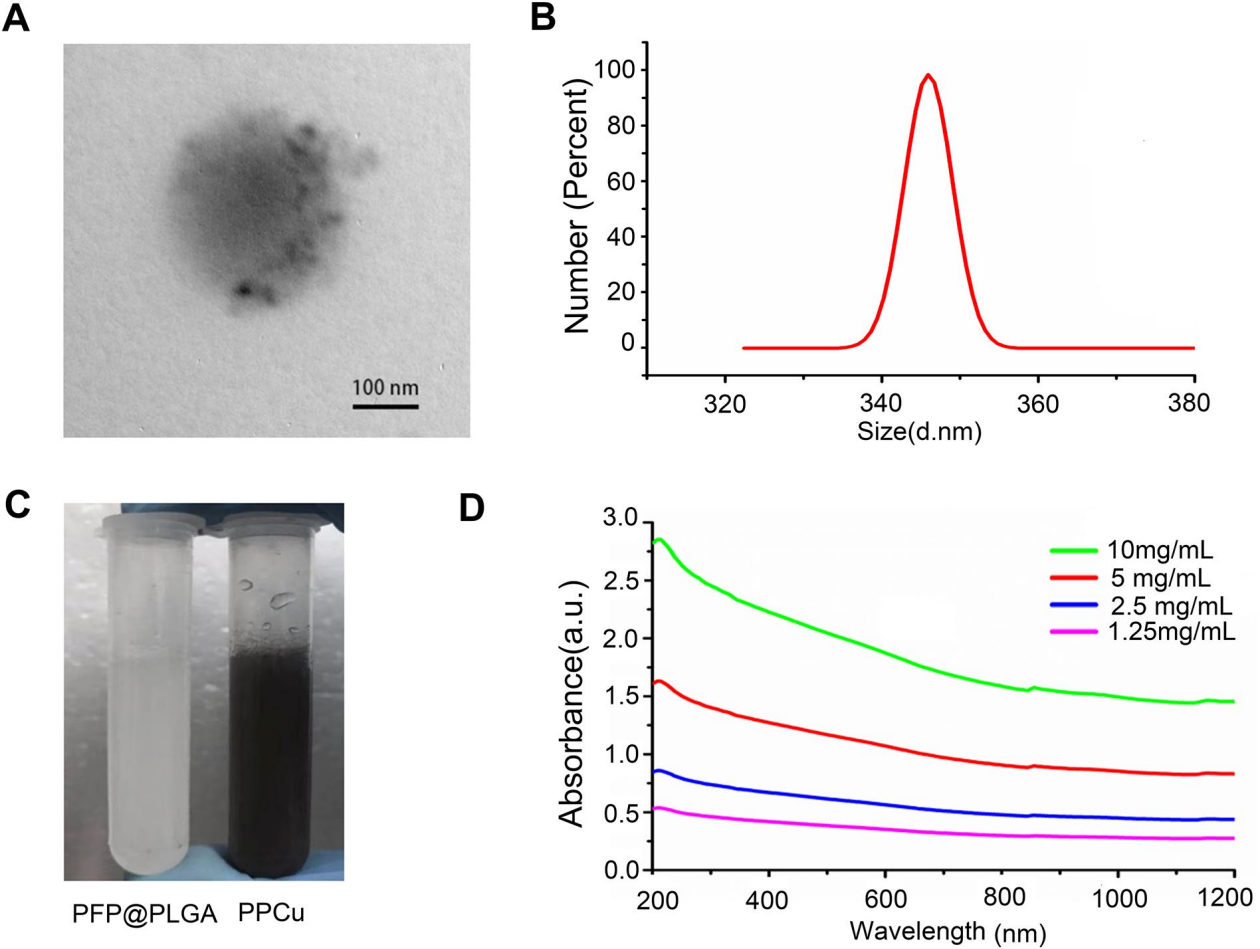
Preparation and characterization of the new PTT agent. **A** TEM of PPCu. **B** Size distribution of PPCu. **C** Photographs of PFP@PLGA and PPCu dispersed in deionized water. **D** UV to NIR absorbance of PPCu in different concentrations

To accomplish US imaging-guided PTT for HCC treatment, PPCu synthesis was realized through a double emulsion evaporation process, in which Cu_12_Sb_4_S_13_, PLGA and PFP were introduced to a reaction mixture in different steps. The transmission electron microscopy (TEM) images revealed that the PPCu and Cu_12_Sb_4_S_13_@PLGA nanoparticle were monodispersed in a spherical shape (Fig. 1A and Additional file 1: Figure S1). The average diameter of PPCu was 346 nm (polydispersity index, PDI = 0.276) (Fig. 1B), and the average surface zeta potential was determined to be − 6.35 ± 2.54 mV. Figure 1C shows that PFP@PLGA nanoparticles and PPCu were white and charcoal grey, respectively, when dissolved in deionized water. The PPCu exhibited broad absorption, ranging from the UV to the near-infrared (NIR) region (Fig. 1D).

### In vitro phase transition and CEUS of the PPCu

**Fig. 2:**
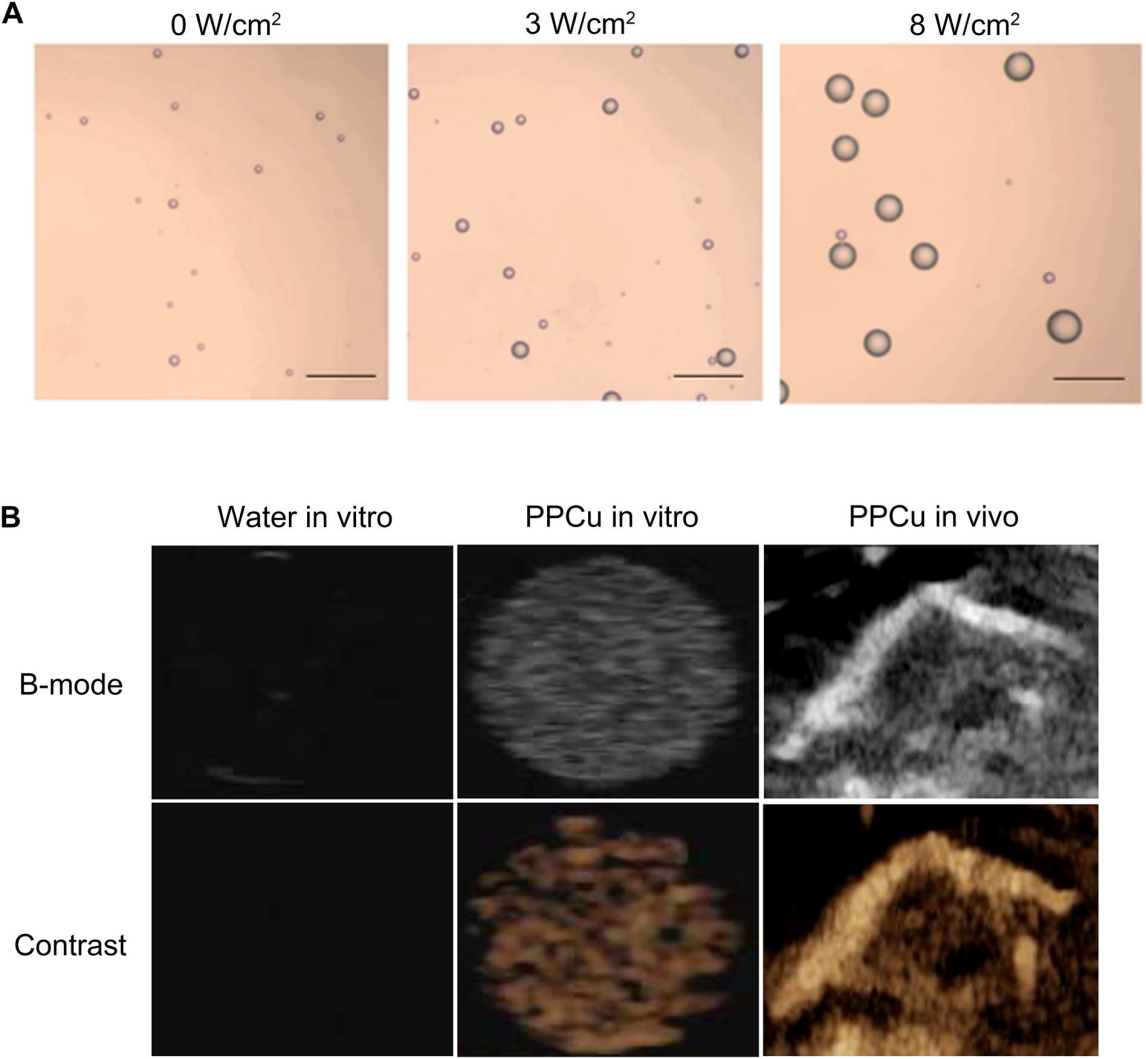
Acoustic droplet vaporization and CEUS of PPCu. **A** Optical microscopic images of PPCu after LIFU treatment with different intensities (0 W/cm^2^, 3 W/cm^2^ and 8 W/cm^2^ for 3 min). The scale bar is 5 μm. **B** B-mode and CEUS mode images of degassed water and PPCu in vitro and in vivo after LIFU stimulation

To confirm the ability of PFP to undergo a phase transition from a liquid to gas state, PPCu stimulated by a LIFU instrument was observed under an optical microscope. As shown in Fig. 2A, the PPCu were clearly enlarged when exposed to 8 W/cm^2^ of US for 3 min, compared to their appearance upon exposure to 3 W/cm^2^ or 0 W/cm^2^. Based on these results, 8 W/cm^2^ of US was applied to stimulate the PPCu for CEUS. The PPCu in the gel mold exhibited high contrast and B-mode imaging performance, but no US signal was detected in the control group (degassed water group). The ADV effect caused by the LIFU instrument induced the PFP phase transition from liquid to gas in the core of the PPCu, which generated more harmonic echo. The effect of CEUS was enhanced with the increase in harmonic waves. Similar results were further obtained in vivo (Fig. 2B).

### Photothermal properties and cytotoxicity of the PPCu

**Fig. 3:**
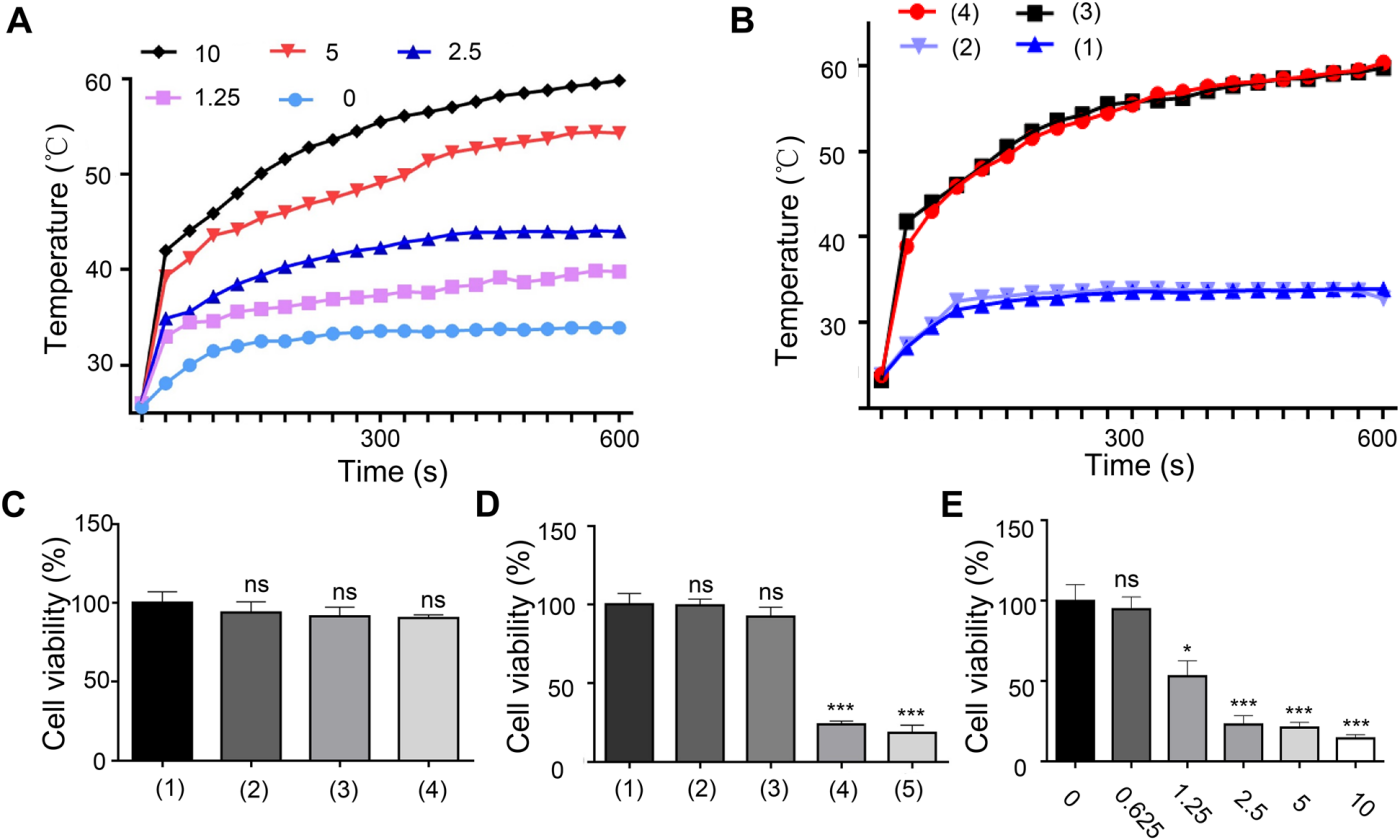
Photothermal properties and cell cytotoxicity of PPCu-mediated PTT. **A** Temperature elevation levels of PPCu in different concentrations (mg/mL) under NIR laser irradiation. **B** Temperature changes of different nanocapsule formulations with NIR laser irradiation. (1), (2), (3), and (4) representing H_2_O@PLGA + NIR, PFP@PLGA + NIR, Cu_12_Sb_4_S_13_@PLGA + NIR, and PPCu + NIR, respectively. **C** Relative cell viability of HepG2 cells treated with DMEM or different nanocapsules. (1), (2), (3), and (4) represent DMEM, PFP@PLGA, Cu_12_Sb_4_S_13_@PLGA and PPCu-treated cells, respectively. **D** Relative cell viability of HepG2 cells treated with different nanocapsule formulations under NIR laser irradiation. (1), (2), (3), (4), and (5) represent DMEM, NIR, PFP@PLGA + NIR, Cu_12_Sb_4_S_13_@PLGA + NIR and PPCu + NIR-treated cells, respectively. **E** Relative cell viability of HepG2 cells treated with different concentrations of PPCu. The x axis represents the concentration in mg/mL. Data are presented as the mean ± SD of three independent experiments. *compared with the control, *p* < 0.05. ** compared with the control, *p* < 0.01. *** compared with the control, *p* < 0.001

Temperature changes in PPCu in response to laser irradiation were explored. The PPCu concentrations tested were 0 mg/mL, 1.25 mg/mL, 2.5 mg/mL, 5 mg/mL and 10 mg/mL. Upon irradiation with 808 nm NIR light at an intensity of 1 W/cm^2^, the temperature of the PPCu rapidly increased (Fig. 3A). In addition, the temperature of the PPCu evelated with increased NIR intensity (Additional file 1: Figure S2), indicating excellent photothermal performance with intensity and concentration-dependent temperature patterns. Upon laser irradiation at the same intensity received by the PPCu, the H_2_O@PLGA nanoparticles in the control group exhibited only a slight temperature increase, up to 33.9 °C. The maximum temperatures of the Cu_12_Sb_4_S_13_@PLGA nanoparticles and PPCu were 59.8 °C and 60.4 °C, respectively, which were significantly higher than those of the H_2_O@PLGA and PFP@PLGA nanoparticles (Fig. 3B). Furthermore, according to an MTT assay, no cytotoxicity was induced by PPCu in cells unexposed to NIR (Fig. 3C). However, upon laser irradiation, HepG2 cells incubated with Cu_12_Sb_4_S_13_@PLGA nanoparticles and PPCu exhibited lower survival rates than the control cells (Fig. 3D). In addition, we measured the mortality rate of different nanocapsule concentrations and found that cell death was induced in a concentration-dependent manner (Fig. 3E). These experiments revealed that the PPCu exhibited a satisfactory light-harvesting ability, which led to the repression of HCC cell proliferation.

### PPCu-mediated PTT ablates HCC tumors in vivo

**Fig. 4:**
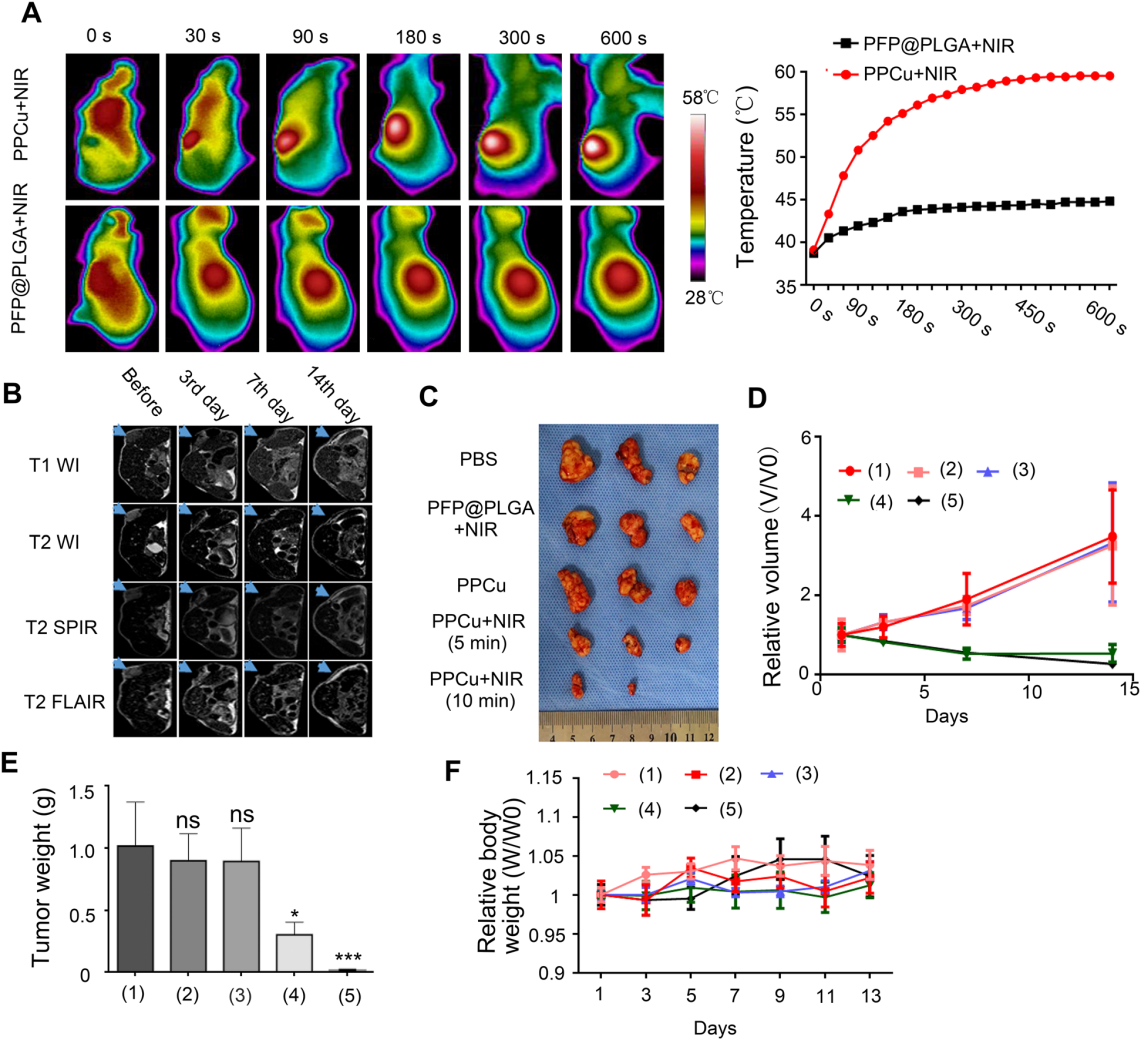
PPCu-mediated PTT ablates HCC tumor in vivo. **A** Thermographic images and corresponding temperature levels of tumor regions under NIR laser irradiation within 10 min. **B** MRI of mice with tumors before and after treatment at the 3rd day, 7th day and 14th day, the arrows indicating the location of tumors. **C** Photographs of excised tumors from mice with different treatments at the 14th day. **D** Relative volume of tumors before and after treatments. (1), (2), (3), (4), and (5) represent PBS, PFP@PLGA + NIR for 10 min, PPCu, PPCu + NIR irradiation for 5 min, and PPCu + NIR irradiation for 10 min, respectively. **E** Average weights of tumors at the 14th day after treatment. (1), (2), (3), (4), and (5) represent PBS, PFP@PLGA + NIR for 10 min, PPCu, PPCu + NIR irradiation for 5 min, and PPCu + NIR irradiation for 10 min, respectively. **F** Relative body weight of mice before and after different treatments. (1), (2), (3), (4), and (5) represent PBS, PFP@PLGA + NIR for 10 min, PPCu, PPCu + NIR irradiation for 5 min, and PPCu + NIR irradiation for 10 min, respectively. Data are presented as the mean ± SD of three independent experiments. *compared with the control, *p* < 0.05. ** compared with the control, *p* < 0.01. *** compared with the control, *p* < 0.001

The capacity of PPCu-mediated PTT was then evaluated in vivo. HepG2 cells were subcutaneously injected into the left flank of nude mice, which had been randomly assigned into five groups (n = 3 mice in each group). When tumor sizes reached approximately 200 mm^3^, 150 µL of PBS, PFP@PLGA or PPCu (5 mg/mL) solution was intratumorally injected into the mice. The five groups are treated as follow: Group 1, PBS; Group 2, PFP@PLGA nanoparticles + NIR irradiation for 10 min; Group 3, PPCu, Group 4, PPCu + NIR irradiation for 5 min; Group 5, PPCu + NIR irradiation for 10 min. An 808 nm NIR laser with an intensity of 0.8 W/cm^2^ was applied to the groups 3 h after injection. A thermometrograph was used to record the temperature changes. As shown in Fig. 4A, the temperature rose rapidly and consistently, to 59.6 °C, at the tumors treated with PPCu + NIR for 10 min and was slight increases upon treatment with PFP@PLGA + NIR. Moreover, MRI, including the T1WI, T2WI, T2-SPIR and T2-FLAIR sequences, was performed to track tumor size dynamically in vivo (Fig. 4B). The tumors in the PPCu + 10 min NIR group shrank or disappeared by the 14th day of treatment (Fig. 4C). The phototherapeutic efficiency was measured through the calculation of tumor volume: V = 1/2 length × width^2^. The relative change in tumor volume was calculated as V/V0 (V0 represents the initial tumor volume), and the average weight was also recorded (Fig. 4D and E). Similarly, W/W0 represents the relative body weight change, and W0 represents the initial weight of the mouse. During the treatment, no obvious change in mouse weight was detected in any group (Fig. 4F), indicating minimal toxicity under the physical conditions. In agreement with the in vitro therapeutic effects measured by MTT assay, a superior antitumor outcome was achieved for the mice receiving PTT, as indicated by all the tumors either shrinking or disappearing after PPCu + NIR treatment, and the effect of NIR exposure for 10 min was treater than that for 5 min.

### Hyperthermia inhibits the proliferation and induces the apoptosis of HCC cells in vitro

**Fig. 5:**
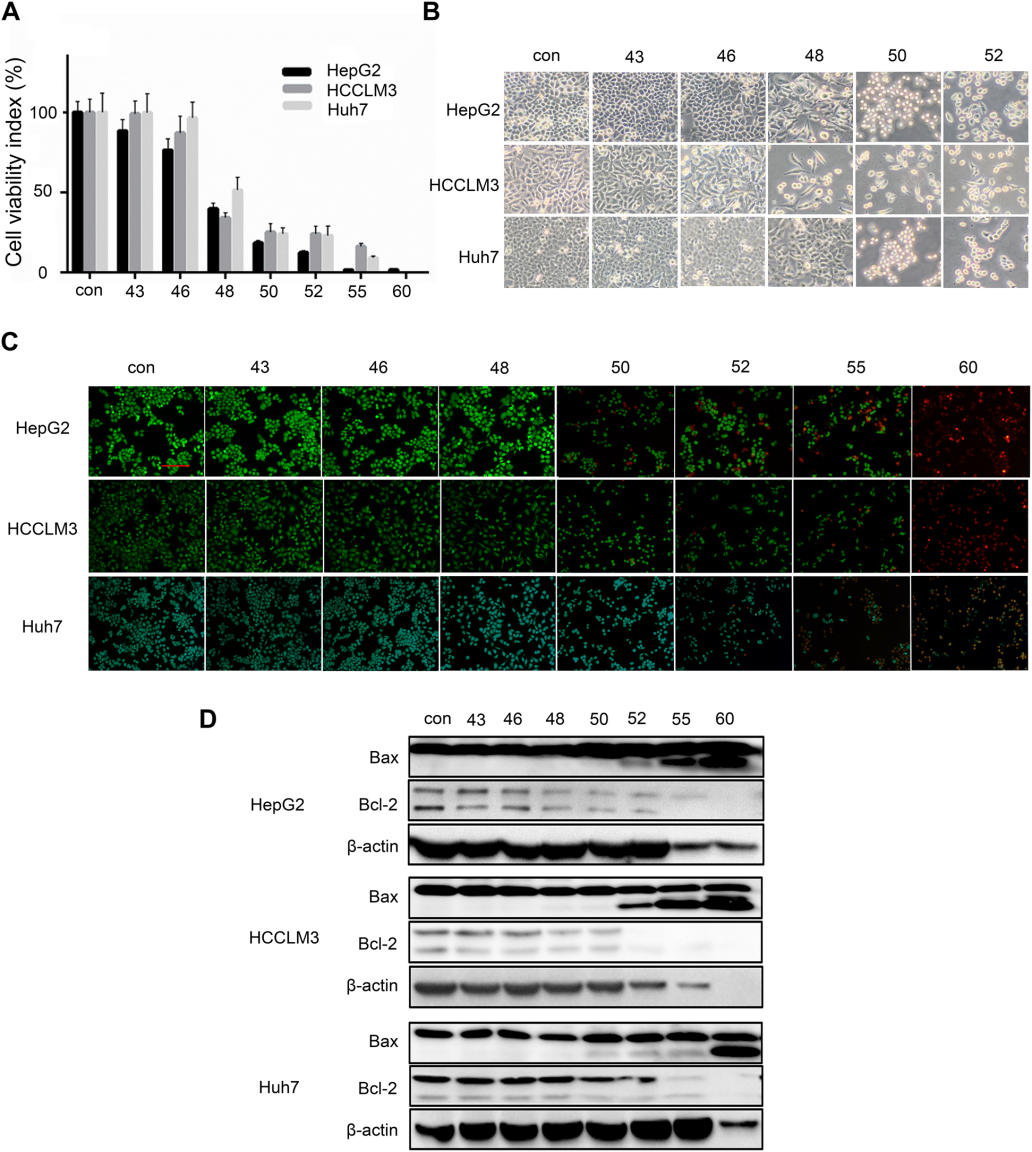
Hyperthermia inhibits proliferation and induces apoptosis in HCC cells in vitro. **A** Cell viability of HepG2, HCCLM3 and Huh7 cells analyzed by MTT assays with increasing gradients of hyperthermia (43–60 °C). **B** Representative cell morphology images of HepG2, HCCLM3 and Huh7 cells with hyperthermia treatment. **C** Representative images of Calcein AM and PI costained cells by fluorescence microscope. **D** The expression of Bax and Bcl-2 explored by western blotting with β-actin used as a control. Data are presented as the mean ± SD of three independent experiments

Photothermal therapy (PTT) refers to nanoparticles embedded within tumors that generate localized heat in response to exogenously applied lasers for the destruction of tumors [30, 31]. Although various PTAs have been used to diminish neoplasms through hyperthermia, the mechanisms of these agents are similar. However, research into the PTT mechanism is hard to achieve due to the difficulty in setting precise temperature gradients. In view of this, we used a water bath model to simulate PTT and followed a preconditioning protocol inducing hyperthermic conditions for 10 min. We first investigated the potential effect of hyperthermia on three HCC cell lines (HepG2, HCCLM3 and Huh7 cells). After treating these cells with increasing hyperthermia-inducing temperature gradients (43–60 °C) for 48 h, the percentage of OD reduction obtained by MTT assay was used as the indicator of cytotoxicity. The results showed that the viability of these HCC cells was diminished with increasing temperature (Fig. 5A), indicating temperature-dependent suppression of cellular viability. A dramatic drop in viability was observed at temperatures higher than 50 °C. The cytotoxic effect of hyperthermia was also confirmed by cell morphology image assessment. The cells underwent transformation into spheres when treated at temperatures higher than 50 °C (Fig. 5B). Based on these results, the effect of hyperthermia was also assessed through fluorescence visualization after performance of a live–dead assay. To this end, Calcein AM, emitting green fluorescence, was used to label living cells, and PI, emitting red fluorescence, was used to label dead cells. Only green fluorescence was detected in the control, 43 °C, 46 °C and 48 °C groups, but when the temperature was higher than 50 °C, the number of dead cells was sharply increased (Fig. 5C).

Apoptosis is a vital process for the inhibition of cancer cell proliferation. Hence, flow cytometric analysis was performed to measure the apoptosis rate of HCC cells treated with increasing hyperthermia-inducing temperature gradients, revealing that the percentage of HCC cells increased in a temperature-dependent manner (Additional file 1: Figure S3). The proportions of apoptotic HepG2, Huh7 and HCCLM3 cells at 52 °C were 40.2%, 28.3% and 24.5%, respectively. Interestingly, most HCCLM3 and HepG2 cells exposed to 55 °C and 60 °C hyperthermic conditions remained in the early apoptosis stage but Huh7 cells similarly treated remained in the late apoptotic or necrotic stage. Furthermore, we measured the levels of Bax and Bcl-2 in HCC cells because they play crucial roles in apoptosis. A western blot analysis indicated that in HepG2, Huh7 and HCCLM3 cells exposed to hyperthermic conditions, the expression of the antiapoptotic protein Bcl-2 was decreased and the expression of the apoptotic protein Bax was increased with increasing hyperthermia-inducing temperature gradients (Fig. 5D). Collectively, these results suggested that hyperthermia inhibited HCC cell proliferation by promoting apoptosis.

### Hyperthermia inhibits the tumorigenesis of HCC in vivo

**Fig. 6:**
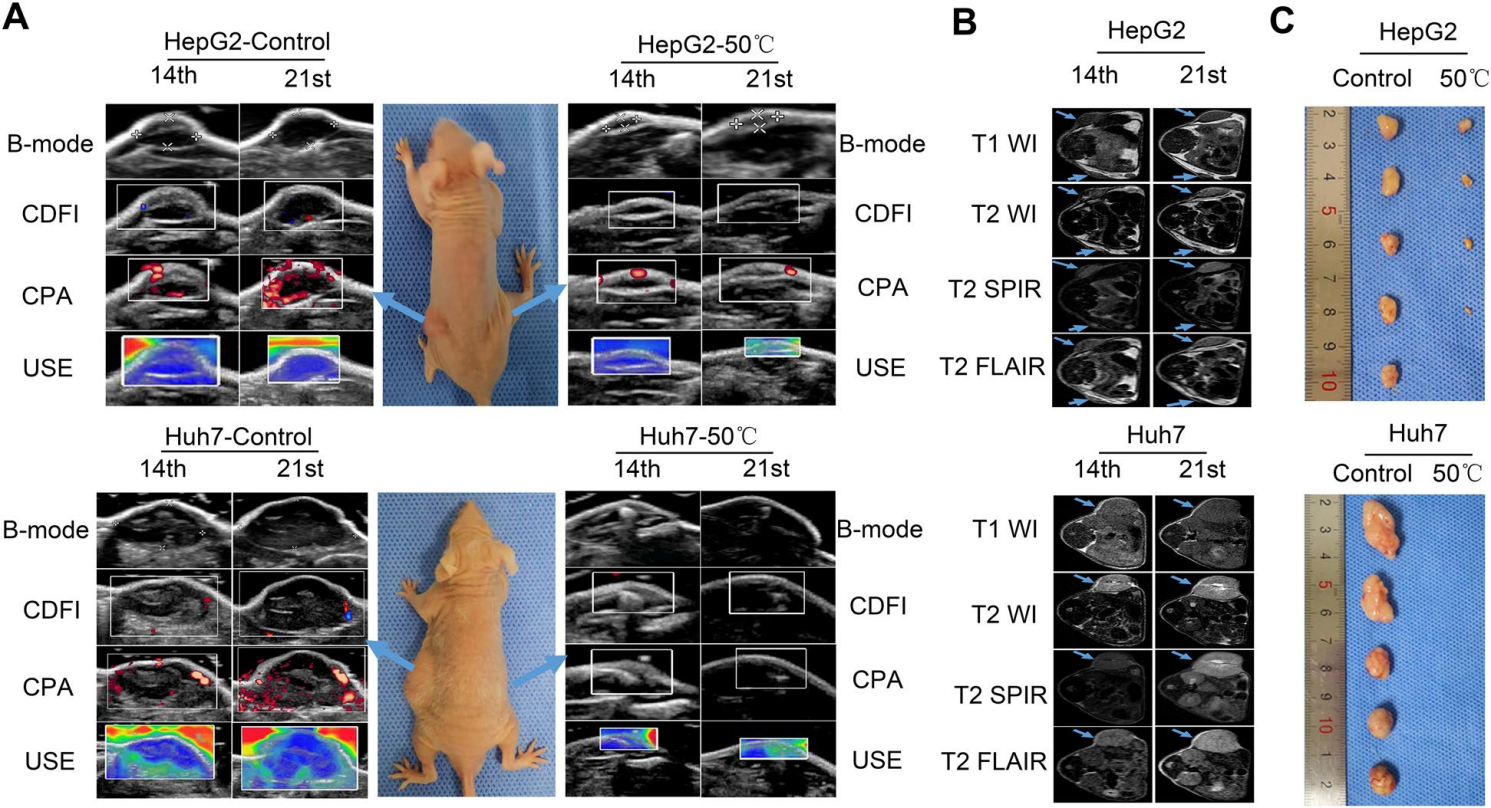
Hyperthermia inhibits growth of HCC in vivo. **A** Representative images of tumors implanted with HepG2 and Huh7 cells by US imaging at the 14th and 21st day. (Left panel represents the control and right panel represents the 50 °C treatment, and arrows indicate US imaging of the corresponding tumors). **B** Representative images of tumors by MRI at the 14th day and 21st day. The arrows indicating the location of tumors. **C** The photographs of excised tumors at the 21st day

Encouraged by the results thus far, the effect of hyperthermia in vivo was explored. Subcutaneous tumor models were established in nude mice by injection with hyperthermia-exposed HepG2 and Huh7 cells. On the 14th and 21st days of the experiment, tumors were noninvasively imaged by US imaging and MRI. Ultrasonography, including B-mode imaging, CDFI, CPA, and USE, was performed to track the size, blood flow volume, energy supply and stiffness of tumors dynamically in vivo. MRI was performed as previously described. The tumors in the HepG2 model mice of the 50 °C hyperthermia group were smaller and exhibited decreased blood flow volume and lower stiffness than the control group. Notably, no tumors were detected in the Huh7 cell-implanted mice in the 50 °C hyperthermia group, indicating the absolute inhibition of HCC expansion upon hyperthermia exposure (Fig. 6A and B). The mice were sacrificed on the 21st day of the experiment, and tumor photographs were taken, and these images revealed results consistent with those obtained by US imaging and MRI (Fig. 6C).

### Hyperthermia inhibits HCC migration and metastasis

**Fig. 7:**
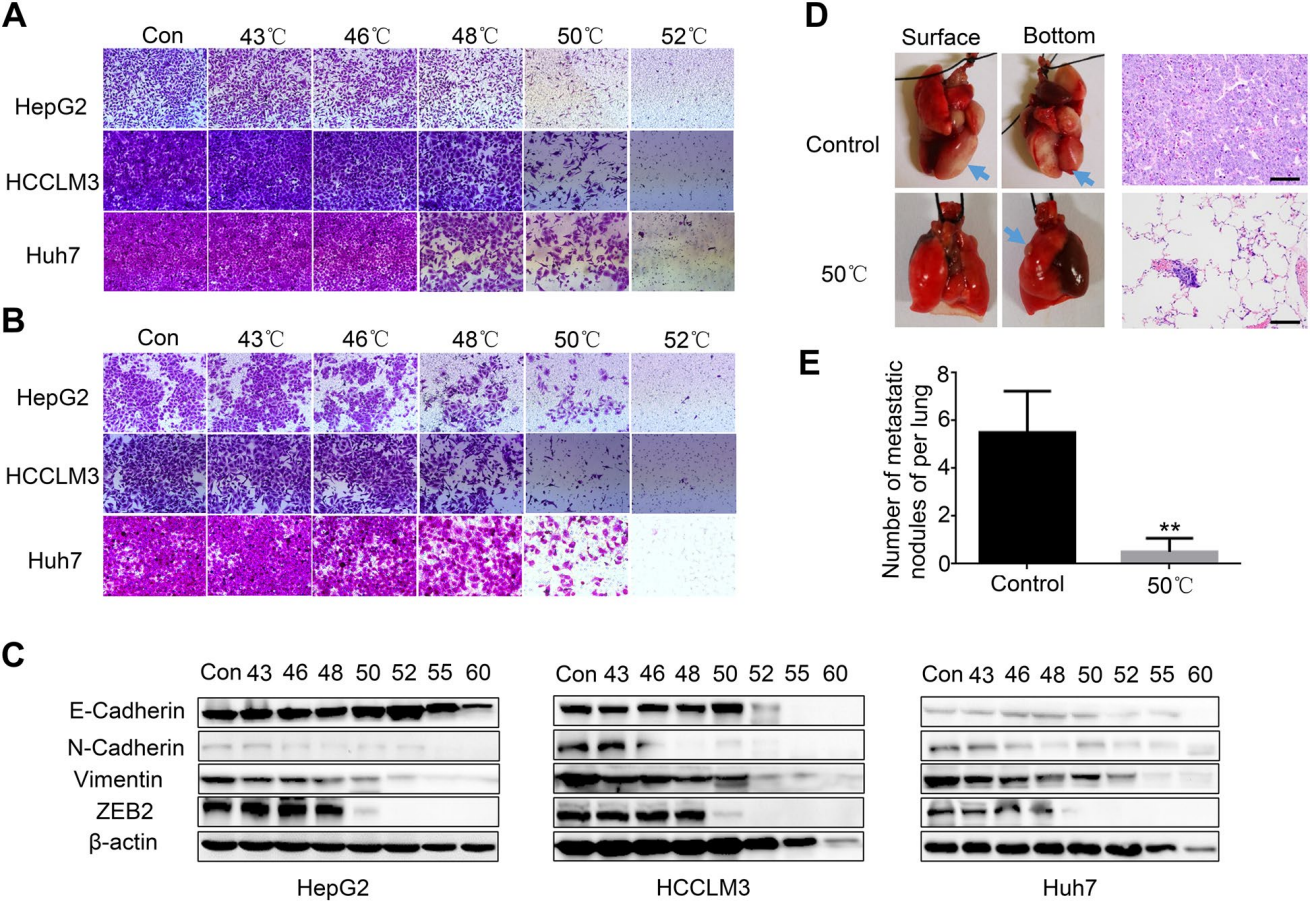
Hyperthermia inhibits HCC migration and invasion in vitro and in vivo. **A** Representative images of migration assays for HCC cells in different groups. **B** Representative images of invasion assays for HCC cells. **C** E-cadherin, N-cadherin, Vimentin and ZEB2 expression evaluated by western blotting in HCC cells. **D** Holistic view and H&E staining of excised lungs from a mouse model of metastasis. Representative images of lung tissues were shown in the left panel. Arrows indicate the location of metastatic lung foci. Corresponding H&E staining of metastatic lung foci were shown in the right panel. The scale bar = 100 μm. **E** Incidence of metastatic lung nodules of each group. **p < 0.01

Migration and invasion, frequently accompanied by the epithelial-mesenchymal transition (EMT), lead to poor survival and high mortality in HCC [32, 33]. Therefore, cell motility was investigated. The migration ability of HepG2, HCCLM3 and Huh7 cells was inhibited in a temperature-dependent manner upon hyperthermia exposure, as determined by Transwell assay (Fig. 7A, Additional file 1: Figure S4A). Hyperthermia exposure also suppressed the invasion ability of these cells in a Matrigel-coated Transwell assay (Fig. 7B, Additional file 1: Figure S4B). Moreover, we measured the expression of the EMT markers E-cadherin, N-cadherin and Vimentin. Among these markers, N-cadherin and Vimentin showed downregulated expression in increasing temperature gradients. However, E-cadherin expression was upregulated at 43–50 °C or 52 °C and then decreased sharply. We also found obvious downregulation of ZEB2 expression in the hyperthermia-exposed HepG2, HCCLM3 and Huh7 cells, thus, ZEB2 a critical EMT-related transcription factor in HCC (Fig. 7C). To further investigate the effects of hyperthermia on HCC metastasis in vivo, we injected HCCLM3 cells with or without exposure to 50 °C hyperthermic conditions into the tail veins of nude mice. Six weeks later, the mice were sacrificed, the lungs were excised, and hematoxylin and eosin staining (H&E) was performed. Fewer and smaller lung metastasis nodules were detected in the 50 °C group than in the control group (Fig. 7D and E). Together, these results demonstrated that hyperthermia inhibits HCC cell migration and metastasis in vitro and in vivo*.*

### Hyperthermia induces mitochondrial dysfunction in HCC cells

**Fig. 8:**
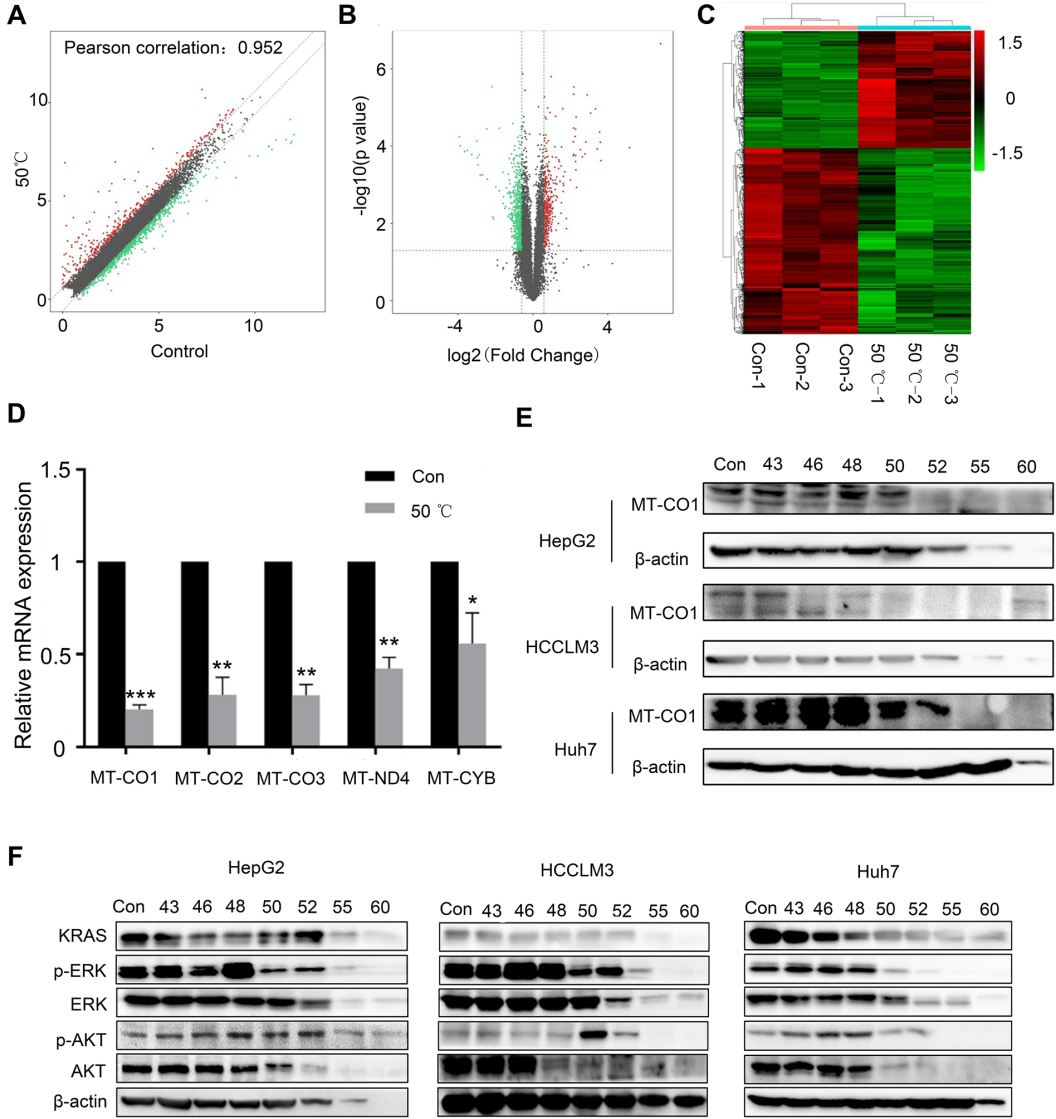
Hyperthermia induces mitochondrial dysfunction of HCC cells. **A** Variation in mRNA expression between the control and 50 °C groups evaluated by scatter plot. The mRNAs above the top dashed line and below the bottom dashed line indicate more than 1.5-fold change. **B** The volcano plot was drawn with differentially expressed genes screened by fold change values and *p*-values. **C** The cluster heat map of differentially expressed mRNAs with low or high expression level indicated by green or red, respectively. **D** Real-time PCR results of top five downregulated mRNAs mentioned above. The results represent three independent tests. *compared with the control, *p* < 0.05. ** compared with the control, *p* < 0.01. *** compared with the control, *p* < 0.001. **E** Expression of MT-CO1 in HepG2, HCCLM3 and Huh7 explored by western blotting and β-actin used as a control. **F** Western blot analysis for the critical regulator of the RAS/MAPK and RAS/PI3K/AKT signal pathways in HepG2, HCCLM3 and Huh7 cells

To further explore the mechanisms by which hyperthermia inhibits the proliferation and migration of HCC cells, we used RNA sequencing to determine which genes levels were altered. The variation in mRNA expression in HepG2 cells with or without 50 °C exposure was revealed in scatter and volcano plots (Fig. 8A and B). In total, 372 mRNAs were upregulated and 589 mRNAs were downregulated in the 50 °C group compared with the control group (Fig. 8C). The 20 mRNAs with the most dramatically downregulated expression are listed in Additional file 1: Figure S5A. A Gene Ontology (GO) analysis indicated that the aforementioned mRNAs were enriched in the regulation of ATP metabolic processes, generation of precursor metabolites and energy, the electron transport chain, etc. (Additional file 1: Figure S6A and B). To validate the results obtained by RNA sequencing (*p* < 0.05), we performed qRT-PCR to confirm the expression of the top five predicted mRNAs with downregulated expression: MT-CO1, MT-CO2, MT-CO3, MT-ND4 and MT-CYB, the results of which were consistent with those obtained by RNA sequencing (Fig. 8D). Among these genes, MT-CO1 encoding cytochrome c oxidase (complex IV), exhibited the most dramatically decreased expression. This result was confirmed by western blot analysis (Fig. 8E). Moreover, we observed the change of mitochondrial morphology after 50 °C treatment for 18 h, which showed dense particles formed in the mitochondria (Additional file 1: Figure S5B). In addition, a pathway analysis indicated that hyperthermia exposure at 50 °C led to decreased expression and activation of the RAS/MAPK and RAS/PI3K/AKT signaling pathways. Therefore, we measured the expression of pivotal proteins in these signaling pathways by western blotting, and the results confirmed downregulated protein expression, including β-actin expression, in the 55 °C and 60 °C groups because of protein degradation. The overall KRAS expression declined in HCC cells, while that of AKT and ERK1/2 (MAPK3) decreased only when the temperature was higher than 50 °C; in contrast, p-ERK1/2 expression was gradually upregulated when the temperature increased from 43 to 48 °C, and then, its overall expression decreased significantly (Fig. 8F). The reduction in p-AKT expression was irregular. We further explored the relationship between RAS/MAPK and MT-CO1 expression. MAPK inhibition by PD98059 may lead to a decrease in MT-CO1 expression, and MT-CO1 expression was downregulated most dramatically when simultaneously treated with PD98059 and exposed to hyperthermia at 50 °C (Additional file 1: Figure S7).

### Mechanistic analysis of PTT with PPCu

**Fig. 9:**
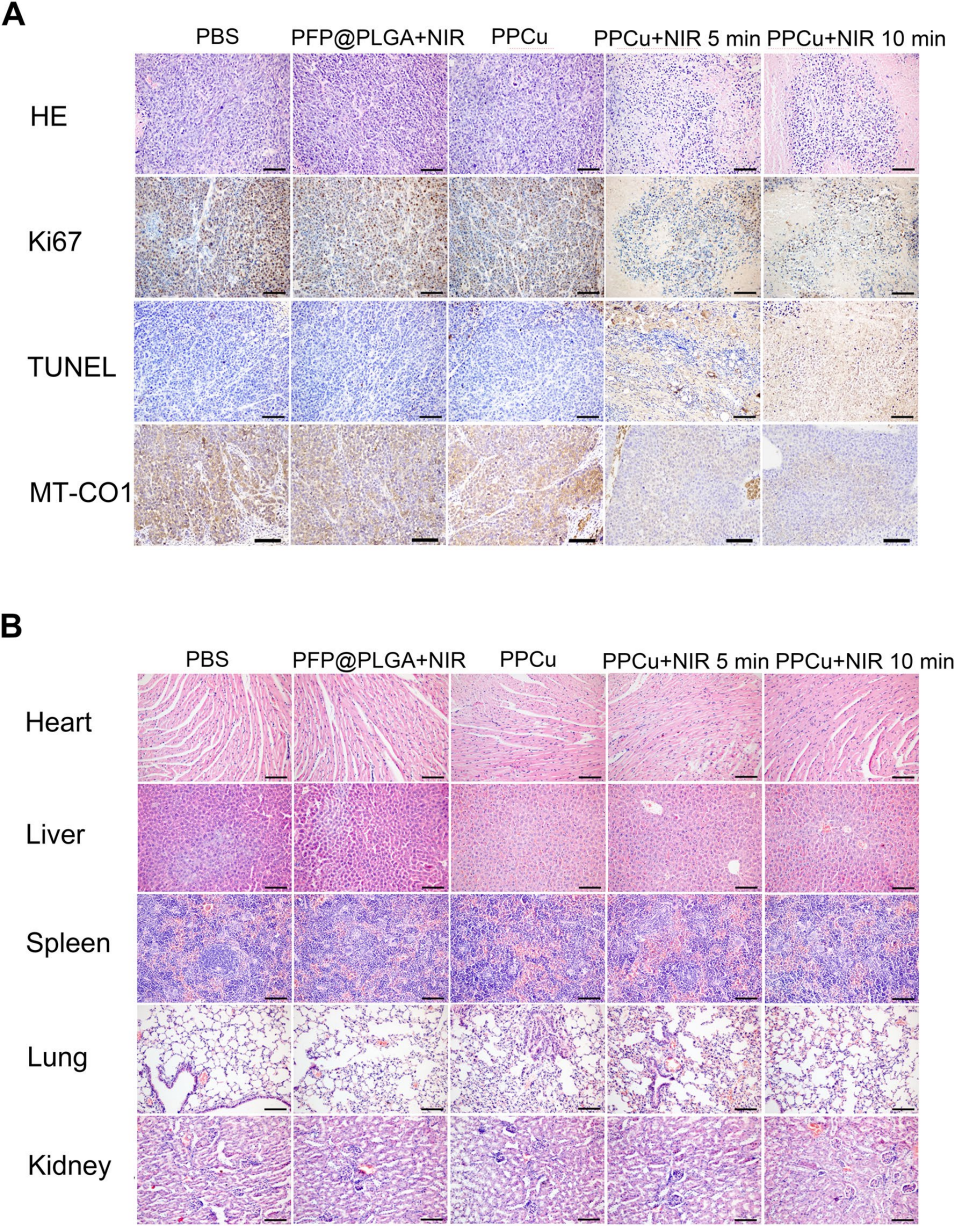
Mechanistic analysis of PTT with PPCu. **A** H&E, ki67, MT-CO1 and TUNEL staining of tumor regions in every group. Scale bar = 100 μm. **B** H&E staining of heart, liver, spleen, lung and kidney collected from different groups of mice at the 14th day after different treatments

Next, we investigated whether PTT achieved with PPCu repressed the growth of HCC by inducing mitochondrial metabolism dysfunction. To this end, histology analysis was performed on the 14th day after PTT with PPCu. H&E staining of tumor tissues revealed that PPCu + NIR treatment for 10 min caused the most significant necrosis of tumor tissues. The expression of MT-CO1 was reduced in the sliced tumor tissues of the PPCu + NIR 10 min group compared with that in the control group. In addition, TUNEL staining indicated that PTT resulted in considerable apoptosis at tumor sites, which was much higher than that in the groups not treated by PTT. Furthermore, cell proliferation inhibition was shown by Ki-67 staining (Fig. 9A). These data were consistent with the results showing tumor regression. The biosafety of nanomaterials is of great significance for their application in the medical field. Considering this, the major organs, including the liver, kidney, heart, lung and spleen, from the mice in all groups were isolated and stained by H&E. As expected, the organ slices showed no obvious variation in H&E staining (Fig. 9B).

## Discussion

PTA can eradicate tumors by rapidly converting NIR light energy into heat, and therefore, these agents have attracted remarkable interest [34]. In recent decades, most of the effort directed to PTA has focused on metal nanomaterials because of their highly effective optical absorption and photothermal conversation. PTT can be initiated when PTA accumulate in tumors through the enhanced permeability and retention (EPR) effect; therefore, it is necessary to determine the distribution of PTA in real time and assess PTT performance on tumors. Nanomaterials amenable to real-time imaging are needed to improve the potential of PTT agents. Ultrasound (US) imaging is a nonradiative, low-cost and portable technology. More importantly, the instant, dynamic and multidimensional features of US imaging render it suitable for locating PTA and observing ablated lesions in real time for precise tumor destruction.

In this research, novel multifunctional nanocapsules (PFP@PLGA/Cu_12_Sb_4_S_13_, PPCu) were synthesized as PTA as well as US contrast agent. The ADV caused by the LIFU instrument enabled the PFP phase transition from liquid to gas in the PPCu core, which generated more echoes in harmonic form for CEUS. Moreover, due to the strong photoabsorption capability of Cu_12_Sb_4_S_13_, the synthesized PPCu were able to generate thermal energy rapidly upon NIR irradiation. Thus, we employed PPCu for CEUS to delineate tumor regions and inhibit tumor growth. Tumor elimination was monitored by MRI to observe the dynamics of tumor progression and elimination.

Elucidating the functional behavior and the mechanism of PTT is essential for improving efficiency and combination therapy options. Because research into the mechanism of PTT is hard to achieve due to the difficulty of setting precise temperature gradients, we first explored the mechanisms of ablation using a water bath. In contrast to traditional hyperthermic conditions, which are set to 37–45 °C for 1 h, we followed a preconditioning protocol for establishing hyperthermia for 10 min, similar to most PTT studies, to imitate clinical thermal ablation operations, which have exposure times of between 1 and 30 min [35–38]. In this study, we evaluated HCC cell survival after exposure to 43–60 °C ablation-inducing temperatures. The survival rate of three HCC cell lines exposed to hyperthermic conditions of 50 °C was merely 20%, which indicated that HCC cells can be killed efficiently upon exposure to hyperthermia at temperatures higher than 50 °C for 10 min. Additional experiments showed that the toxicity induced by exposure to hyperthermia at 50 °C was due to apoptosis and necrosis, similar to previous traditional hyperthermia studies. Moreover, hyperthermia reduced HepG2, Huh7 and HCCLM3 cell invasion and migration without inducing toxicity like radiotherapy and chemotherapy. The EMT program was inhibited, at least partially, leading to invasion and migration inhibition. In vivo, exposure to 50 °C hyperthermic conditions significantly inhibited subcutaneous xenograft tumor growth and lung metastasis in nude mice. These results highlighted the antitumor effect of hyperthermia.

We further investigated the mechanism of hyperthermia function in HCC cells by performing RNA sequencing, and the results revealed that hyperthermia killed HCC cells by reducing the expression of the subunits of multiple electron transport chain complexes. Among the most differentially expressed genes predicted by RNA sequencing in hyperthermia groups compared to the control group, MT-ND1, MT-ND2, MT-ND4, and MT-ND5 are all complex I subunits, and MT-CO1 and MT-CO2 are both complex IV subunits [39–41]. All of these subunits contribute to mitochondrial respiration. The ETC promotes the conversion of ADP to ATP, and elevated cellular energy production is favorable for tumor cell metastasis [42, 43]. MT-CO1 expression was the most drastically reduced, which further disrupts the other complex subunits in the ETC. The downregulation of MT-CO1 was confirmed by qRT-PCR and western blot analyses. Collectively, these data indicated that the expression of subunits of multiple electron transport chain complexes decreases in response to hyperthermic stress. Since the functions of HIF1-α, c-myc, RAS, ERK1/2, PI3K/AKT/mTOR, and IGF-1 are directly or indirectly to subserve the cellular energy infrastructure and because pathway analysis on the basis of genes identified by RNA sequencing in this study revealed that 50 °C hyperthermic conditions can lead to the downregulation of RAS/MAPK, RAS/PI3K/AKT signaling pathway, we measured the critical molecules in both of these pathways. The results showed that the levels of activated ERK1/2, total ERK1/2 and AKT decreased with temperature increases and that the decrease in AKT phosphorylation was irregular. Therefore, our results further revealed that RAS/MAPK signaling pathway inhibition reduced the expression of MT-CO1, which is a subunit component of multiple electron transport chain complexes. These results illustrated that hyperthermia may induce mitochondrial metabolism alterations by affecting the RAS/MAPK/MT-CO1 signaling pathway.

The mechanism by which hyperthermia affects HCC was verified in PTT-treated tumor tissue. Immunohistochemistry and TUNEL assay results indicated that proliferation was inhibited and apoptosis was promoted in PTT-treated tumor tissue. Moreover, the expression of MT-CO1 was decreased, in line with the results obtained from hyperthermia experiments showing that PTT eradicates tumors by suppressing the expression of subunits of multiple electron transport chain complexes. In addition, no obvious injury to major organs, namely, the liver, kidney, heart, lung and spleen, was detected by H&E staining, demonstrating the biosafety of the PPCu.

In this study, novel multifunctional nanocapsules were constructed. Although Cu_12_Sb_4_S_13_ has not been approved by the FDA similar to most new PTAs, the biosafety of PPCu was tested and verified preliminarily through MTT assay in vitro and H&E staining in vivo. In addition, Cu, Sb and S are all essential elements that have been used in many PTT studies [44–46], and we will verify their safety in additional animal studies in the future. In the past, PTT exposed to NIR irradiation was used to treat superficial tumors such as breast cancer due to its minimal penetrating effect. Recently, PTT has been used to heat deep-seated tumors in an increasing number of studies. Improvements in heating devices and optimization of the heating methodology has facilitated better heating of deep-seated tumors. The laparoscopic technique is widely used for minimally invasive surgery in clinical treatment, and it can be used to detect the tumor location visually and precisely, thereby enhancing and assisting the PTA effectiveness in real time. An 18 gauge (G) percutaneous catheter containing an NIR optical fiber was applied to deliver a laser beam deep into tissues and selectively destroyed tumor tissue [47]. Therefore, devices such as laparoscopic-assisted percutaneous catheter can be used for wide application of PPCu in the treatment of deep-seated solid tumors.

## Conclusion

In conclusion, novel multifunctional nanocapsules (PFP@PLGA/Cu_12_Sb_4_S_13_, PPCu) were successfully constructed in this work. We employed PPCu to repress tumor growth and conduct CEUS to delineate tumor regions. Moreover, the mechanism of PTT on HCC was investigated, and the results demonstrated that, at 50 °C and higher temperatures, the cell apoptosis rate was increased and cell motility was decreased because PTT inhibited RAS/MAPK/MT-CO1 signaling pathway. Hence, this work provides a guideline for setting an effective PTT temperature and a breakthrough point for combination therapy, and it expands the possibilities of using nanocarriers as multifunctional nanoplatforms.

## Supplementary Information

**Additional file 1: ** Additional figures (Fig. S1−S7).

## Data Availability

Not applicable.

## Abbreviations

HCC: Hepatocellular carcinoma
HT: Hyperthermia
PTT: Photothermal therapy
PTA: Photothermal transduction agent
PLGA: Poly (lactic-co-glycolic acid)
LIFU: Low intensity focused ultrasound
PPCu: PFP@PLGA/Cu_12_Sb_4_S_13_ nanocapsule
ETC: Electron transport chain
MT-CO1: Cytochrome c oxidase subunit 1
TEM: Transmission electron microscopy
ADV: Acoustic droplet vaporation
US: Ultrasound
CEUS: Contrast-enhanced ultrasound imaging
CDFI: Color doppler flow imaging
CPA: Color power angiography
USE: Ultrasonic elastosonography
PI: Propidium
EMT: Epithelial–mesenchymal transition
GO analysis: Gene ontology analysis
MAPK: Mitogen-activated protein kinase
