# Correction to: PFP@PLGA/Cu_12_Sb_4_S_13_‑mediated PTT ablates hepatocellular carcinoma by inhibiting the RAS/MAPK/MT‑CO1 signaling pathway

**DOI:** 10.1186/s40580-024-00444-3

**Published:** 2024-09-18

**Authors:** Tianxiu Dong, Jian Jiang, Hao Zhang, Hongyuan Liu, Xiaomeng Zou, Jiamei Niu, Yingxuan Mao, Mingwei Zhu, Xi Chen, Zizhuo Li, Yaodong Chen, Chunying Shi, Xiuhua Yang

**Affiliations:** 1 Department of Abdominal Ultrasound, The First Affiliated Hospital of Harbin Medical University, Harbin, 150001 China; 2 Department of Medical Imaging, Heilongjiang Provincial Hospital, Harbin, 150001 China; 3 Department of Ultrasonic Imaging, First Hospital of Shanxi Medical University, Taiyuan, 030001 China; 4 Department of Radiology, The First Affiliated Hospital of Harbin Medical University, Harbin, 150001 China

**Correction to: Nano Convergence (2021) 8:29**

10.1186/s40580-021-00279-2

Following publication of the original article [1], the author identified the errors in the Figures, Supplementary Material and Availability of data and materials. The corrected Figs. 7A, 9A, B and Fig. S3 presented with this correction.

**Fig. 7:**
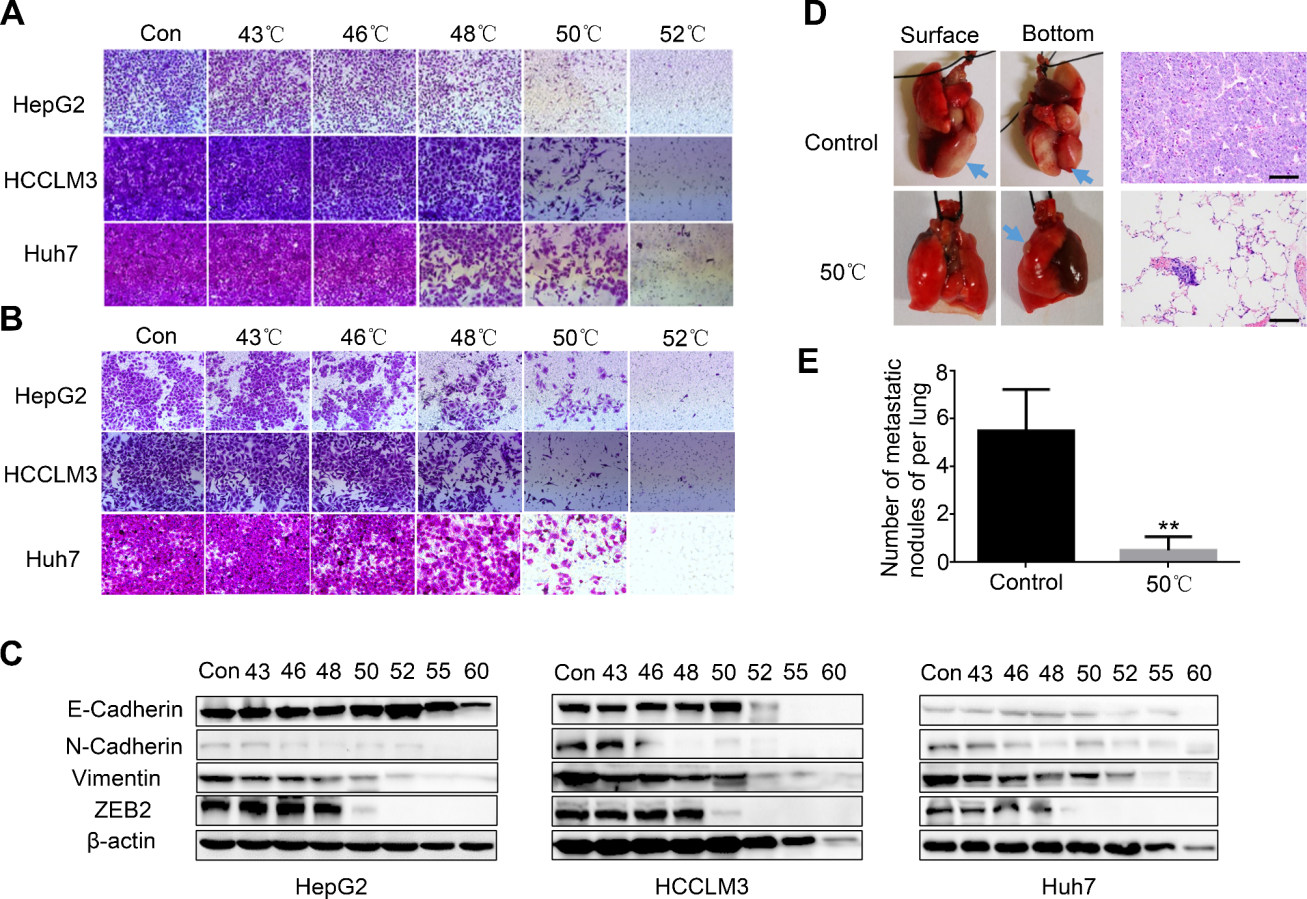
Hyperthermia inhibits HCC migration and invasion in vitro and in vivo. **A** Representative images of migration assays for HCC cells in diferent groups. **B** Representative images of invasion assays for HCC cells. **C** E-cadherin, N-cadherin, Vimentin and ZEB2 expression evaluated by western blotting in HCC cells. **D** Holistic view and H&E staining of excised lungs from a mouse model of metastasis. Representative images of lung tissues were shown in the left panel. Arrows indicate the location of metastatic lung foci. Corresponding H&E staining of metastatic lung foci were shown in the right panel. The scale bar=100 μm. **E** Incidence of metastatic lung nodules of each group. **p<0.01

**Fig. 9:**
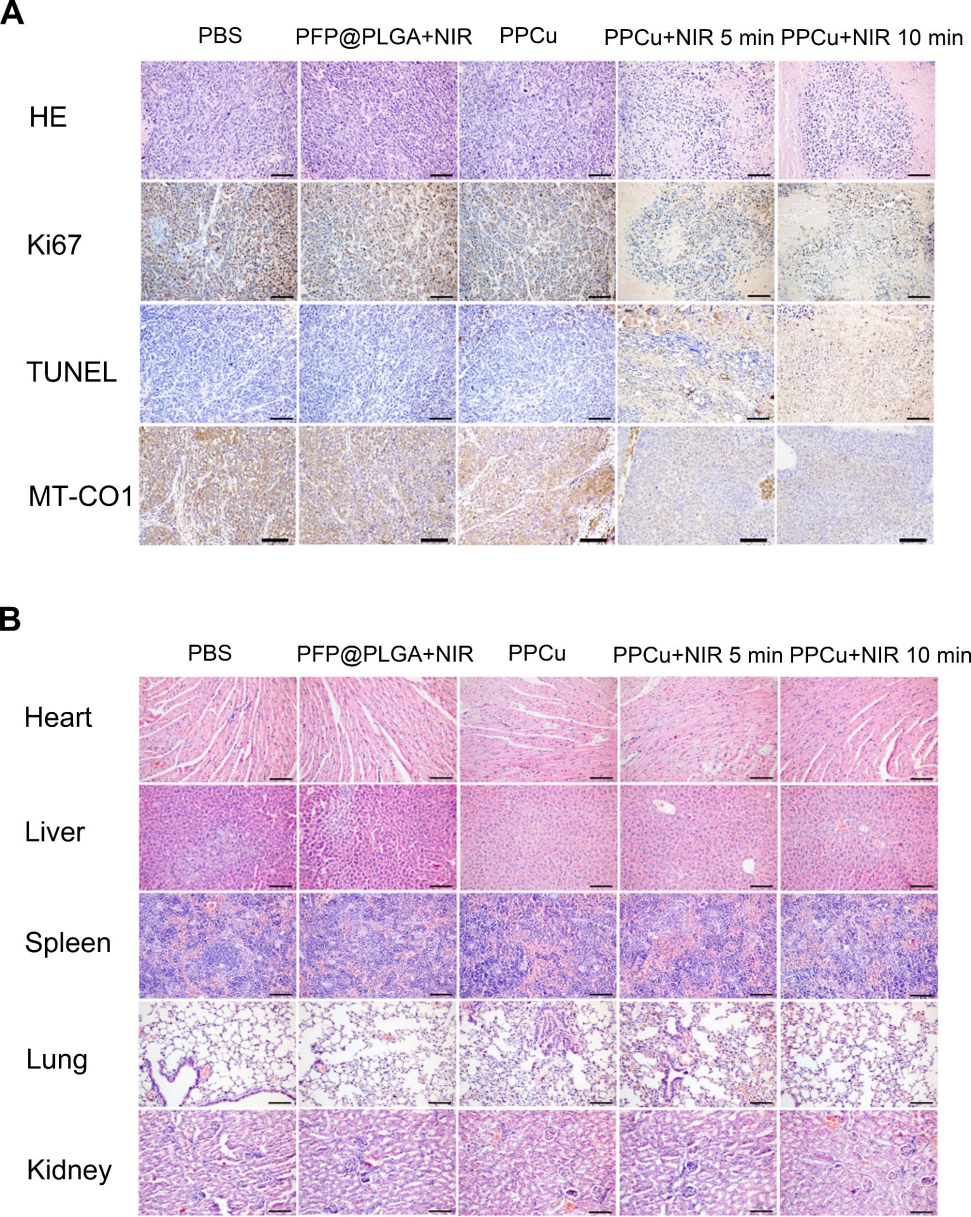
Mechanistic analysis of PTT with PPCu. **A** H&E, ki67, MT-CO1 and TUNEL staining of tumor regions in every group. Scale bar=100 μm. **B** H&E staining of heart, liver, spleen, lung and kidney collected from diferent groups of mice at the 14th day after diferent treatments

## Electronic supplementary material

Below is the link to the electronic supplementary material.

Supplementary Material 1

## Data Availability

All datasets and materials used during the current study are available from the corresponding author on reasonable request.
