# Supplementary material for: Correction to: PFP@PLGA/Cu12Sb4S13‑mediated PTT ablates hepatocellular carcinoma by inhibiting the RAS/MAPK/MT‑CO1 signaling pathway
Source: Nano Converg. 2024 Sep 18;11:37. doi: 10.1186/s40580-024-00444-3 (PMC11411021; doi:10.1186/s40580-024-00444-3)
Supplement: Supplementary file 1 — Supplementary Material 1 [file 40580_2024_444_MOESM1_ESM.docx]

**Additional file 1.**

**
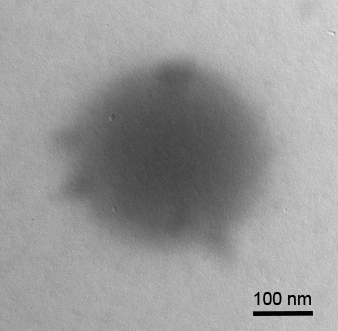
**

**Fig. S1.** TEM of Cu_12_Sb_4_S_13_@PLGA.





**Fig. S2.** Temperature elevation levels of PPCu induced by different intensities of NIR.

**
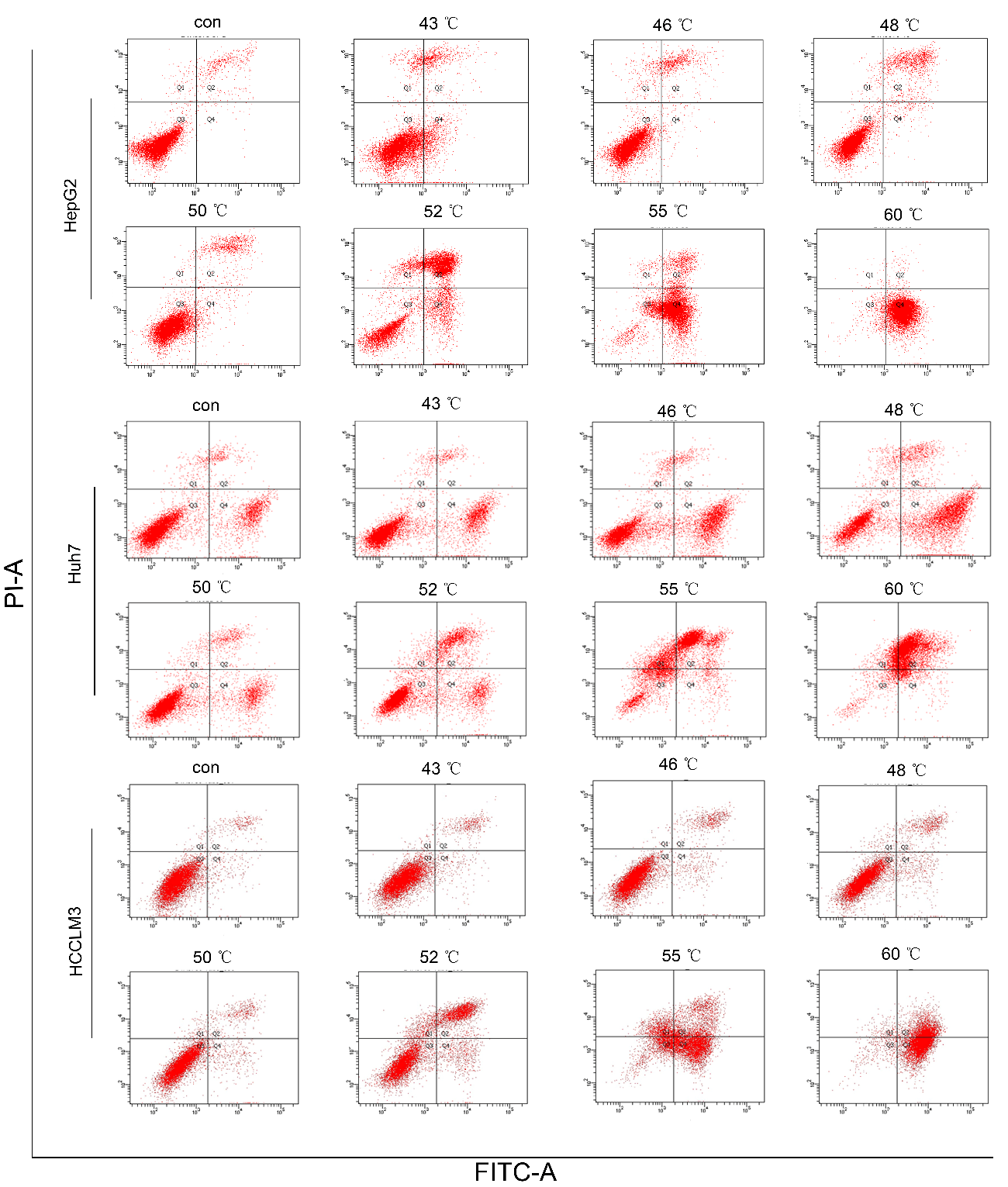
**

**Fig. S****3.** Cell apoptosis detected by flow cytometry for HCC cells treated with increasing temperatures.


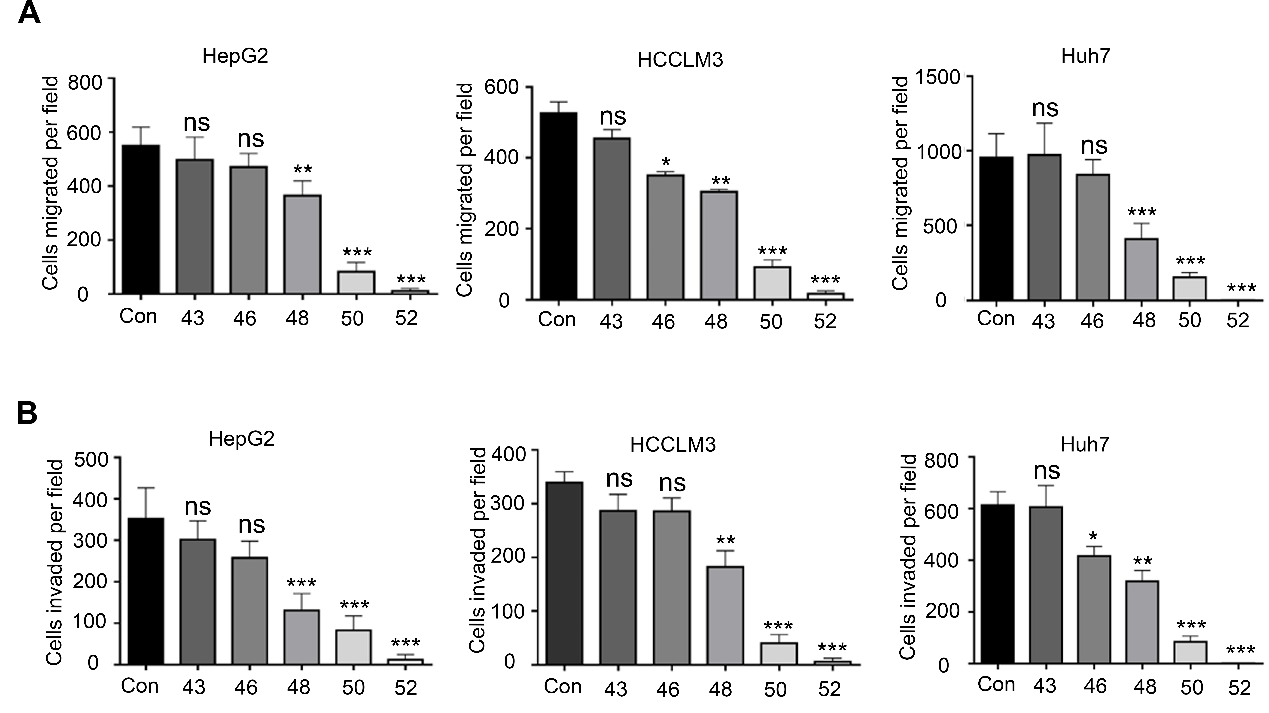


**Fig. S4.** Statistical analysis of cell migration (A) and invasion (B). Data are presented as the mean ± SD of three independent experiments. *compared with the control, *p* < 0.05. ** compared with the control, *p* < 0.01. *** compared with the control, *p* < 0.001.


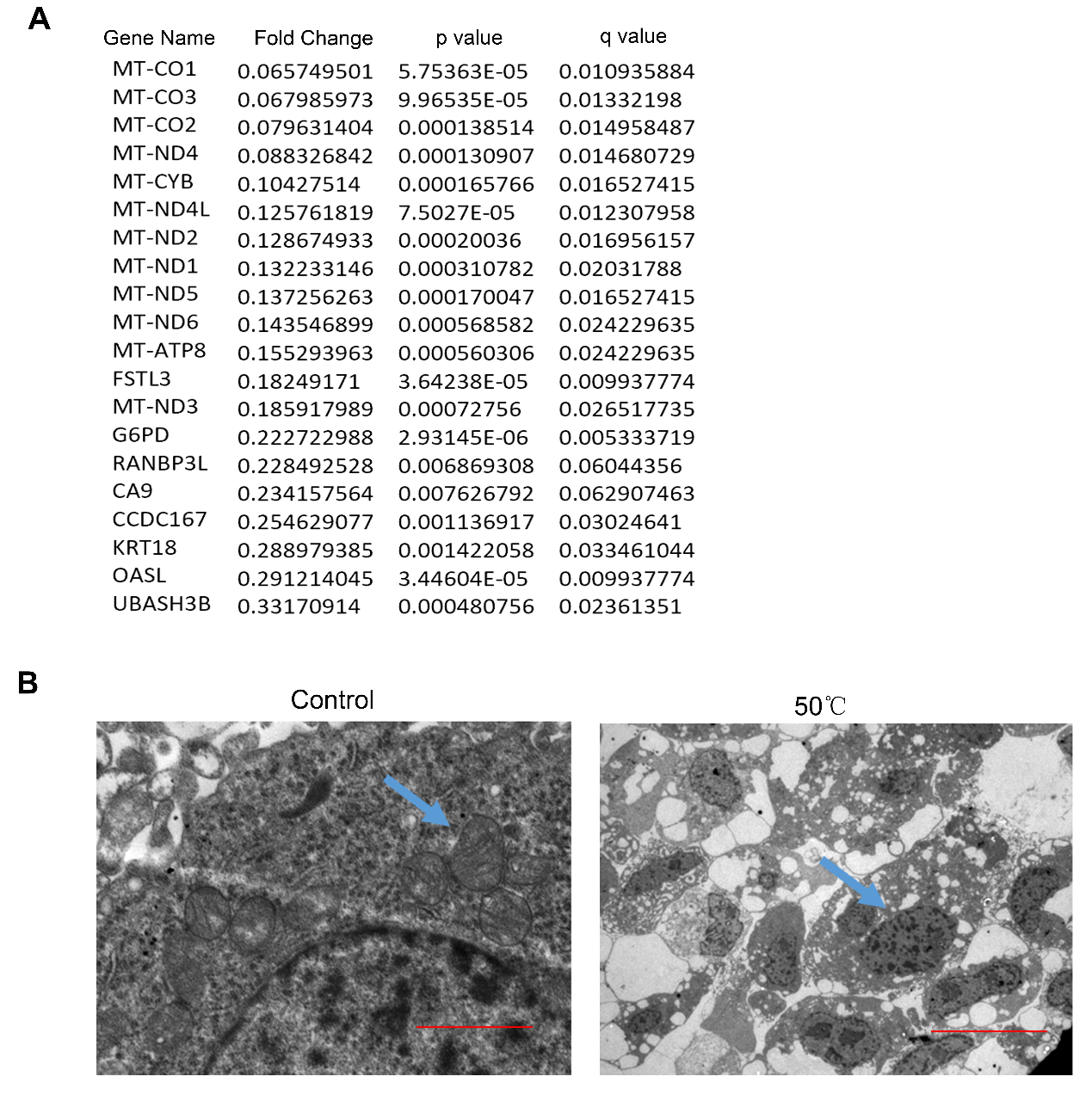


**Fig. S5.** (A) List of top 20 downregulated mRNAs. (B) Representative images of mitochondria changes taken by TEM and arrows indicating mitochondria. The scale bar =1 μm.


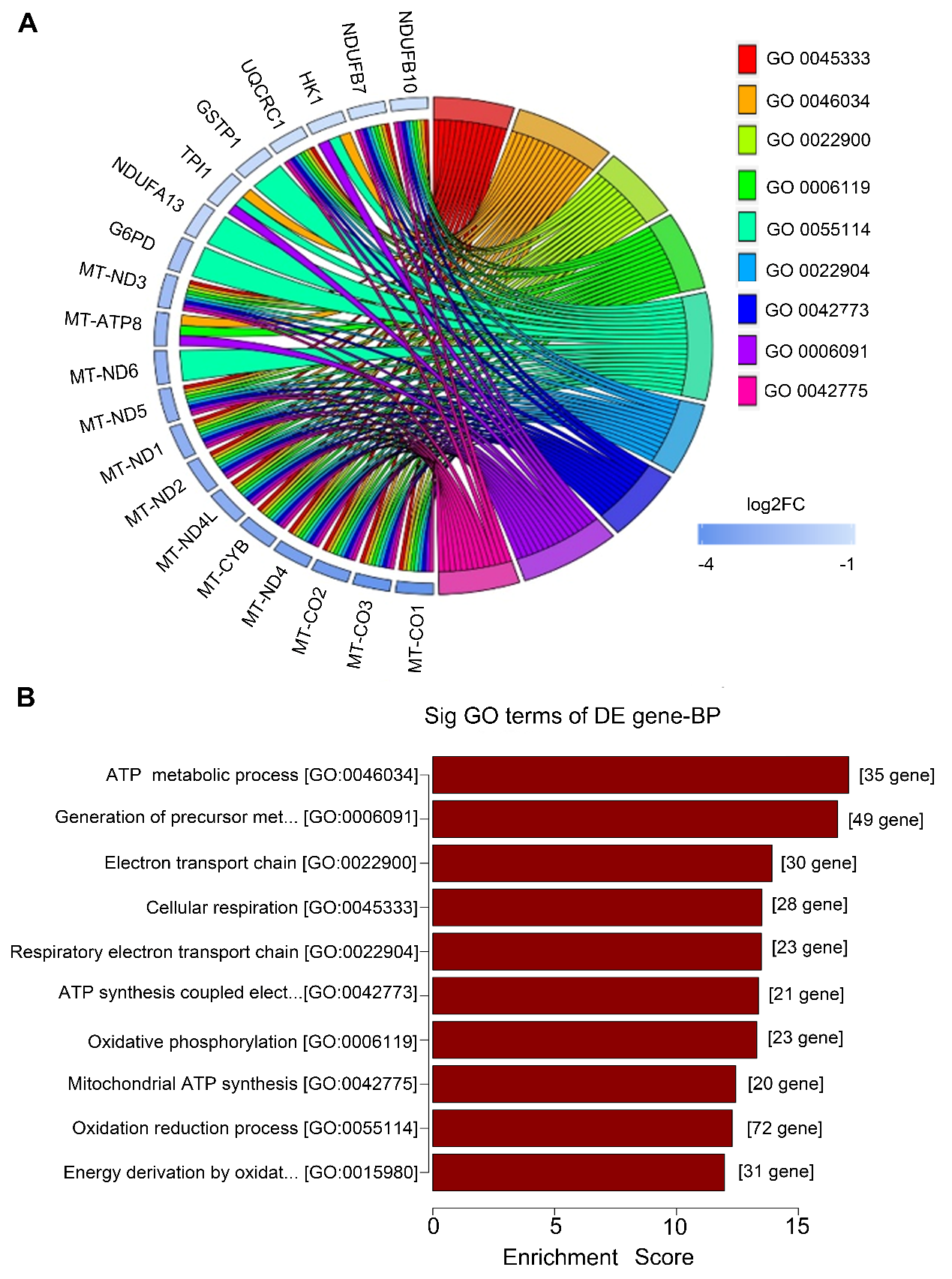


**Fig. S6.** (A) GO indicating molecular function of different genes. (B) GO analysis of the numbers of differentially expressed genes appearing in the corresponding enrichment terms.


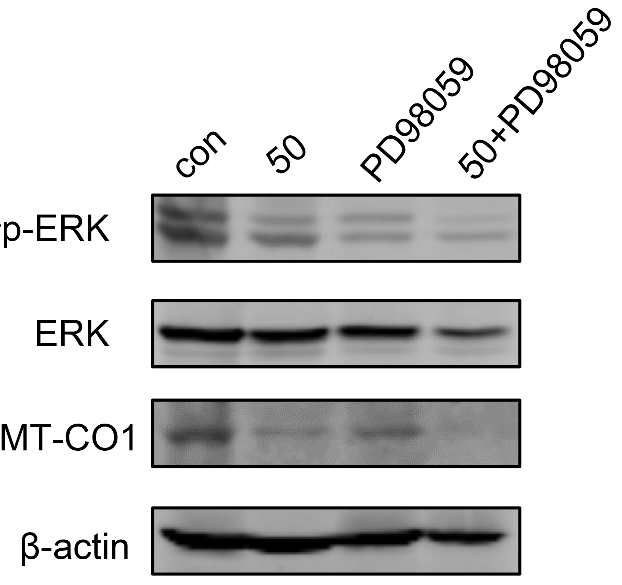


**Fig. S7.** Expression of p-ERK, ERK, MT-CO1, and β-actin evaluated by western blotting in HepG2 cells.
